# Hot Carrier Transport and Carrier Multiplication Induced High Performance Vertical Graphene/Silicon Dynamic Diode Generator

**DOI:** 10.1002/advs.202200642

**Published:** 2022-05-23

**Authors:** Yanghua Lu, Runjiang Shen, Xutao Yu, Deyi Yuan, Haonan Zheng, Yanfei Yan, Chang Liu, Zunshan Yang, Lixuan Feng, Linjun Li, Shisheng Lin

**Affiliations:** ^1^ College of Microelectronics College of Information Science and Electronic Engineering Zhejiang University Hangzhou 310027 P. R. China; ^2^ State Key Laboratory of Modern Optical Instrumentation Zhejiang University Hangzhou 310027 P. R. China

**Keywords:** carrier multiplication, dynamic diode, generator, graphene, hot carrier transport

## Abstract

Dynamic semiconductor diode generators (DDGs) offer a potential portable and miniaturized energy source, with the advantages of high current density, low internal impedance, and independence of the rectification circuit. However, the output voltage of DDGs is generally as low as 0.1–1 V, owing to energy loss during carrier transport and inefficient carrier collection, which requires further optimization and a deeper understanding of semiconductor physical properties. Therefore, this study proposes a vertical graphene/silicon DDG to regulate the performance by realizing hot carrier transport and collection. With instant contact and separation of the graphene and silicon, hot carriers are generated by the rebounding process of built‐in electric fields in dynamic graphene/silicon diodes, which can be collected within the ultralong hot electron lifetime of graphene. In particular, monolayer graphene/silicon DDG outputs a high voltage of 6.1 V as result of ultrafast carrier transport between the monolayer graphene and silicon. Furthermore, a high current of 235.6 nA is generated due to the carrier multiplication in graphene. A voltage of 17.5 V is achieved under series connection, indicating the potential to supply electronic systems through integration design. The graphene/silicon DDG has applications as an in situ energy source for harvesting mechanical energy from the environment.

## Graphical abstract

The vertical graphene/silicon dynamic diode generator (DDG) is proposed and the detailed hot carrier dynamics are explored. High voltage of 6.1 V and current of 235.6 nA are achieved in monolayer graphene/silicon DDG unit, attributing to ultrafast carrier transport and carrier multiplication in graphene. This work provides a novel and potential in situ source for harvesting mechanical energy from the environment.

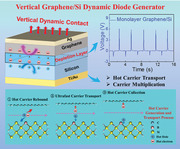

## 1 Introduction

The global development of “carbon neutral” strategies presents new energy demands,^[^ ^1^ ^]^ therefore, urgent development of electric conversion devices for collecting solar energy,^[^ ^2^ ^]^ mechanical energy,^[^ ^3^ ^]^ and tidal energy^[^ ^4^ ^]^ is required. Among these clean energy technologies, mechanical energy is one of the most abundant energy sources with no time and geographical location limitations, and is usually harvested by electromagnetic generators.^[^ ^5^ ^]^ However, electromagnetic generators require electromagnetic windings and permanent magnets, for which miniaturization and wearable integration are major challenges.^[^ ^6^ ^]^ Meanwhile, the recently developed dynamic semiconductor diode generator (DDG) is a good candidate for a miniaturized, portable, integrated, and renewable micro generator, able to harvest low‐frequency mechanical energy.^[^ ^7^ ^]^ Furthermore, the rise of the Internet of Things (IoTs) and wearable electronic devices presents new demands for in‐situ energy and sensors, including miniaturization, high power density, lightness, integration, portability, and flexibility.^[^ ^8^ ^]^ The DDG has significant advantages that meet the requirements of IoTs devices, including high current density, low internal impedance matching with semiconductor based electron devices, no storage or external circuit restrictions, and continuous and stable power generation.^[^ ^9^ ^]^

The structure and physical characteristics of DDGs have been widely explored, including the dynamic metal‐semiconductor,^[^ ^10^ ^]^ semiconductor‐semiconductor,^[^ ^11^ ^]^ metal‐semiconducting polymer,^[^ ^12^ ^]^ and liquid‐semiconductor structure generators.^[^ ^13^ ^]^ However, the voltage performance of vertical DDGs is inferior owing to energy loss during carrier transport and collection, which is usually as low as 0.1–1V and requires further enhancement for wider applications.^[^ ^14^ ^]^ Under the semiconductor physics framework, the electrical energy conversion phenomenon is attributed to the dynamic equilibrium of the semiconductor depletion region and carrier rebounding at the interface during mechanical movement.^[^ ^7^ ^]^ The rebounding electrons and holes in the dynamic semiconductor depletion region are separated and accelerated by the built‐in electric field at the interface, generating hot carriers with high energy.^[^ ^15^ ^]^ However, owing to the energy loss during the carrier transport and inefficient carrier collection in the semiconductor, high energy hot carriers excited by mechanical input and the built‐in electric field are difficult collect effectively in traditional DDG structures, leading to an inferior voltage and current performance. In contrast to the accumulated charges in polymer‐based generators, rebounded hot carriers in semiconductor‐based generators can instantaneously rebound and accelerate from the interface to electrodes. However, it remains a challenge to collect and utilize the rebounding hot carriers in dynamic semiconductor diodes, which is vital to enhance the performance of DDGs. Furthermore, the detailed carrier dynamics of a dynamic semiconductor junction require further detailed investigations, including the establishment, dynamic equilibrium and destruction processes.^[^ ^16^ ^]^

As a 2D material, graphene has significant advantages of excellent carrier mobility, a zero band gap, and superconductivity.^[^ ^17^ ^]^ The most attractive attributes of graphene are the ultralong hot electron lifetime and linear energy dispersion, which are attributed to the strong in‐plane electron‐electron interactions.^[^ ^18^ ^]^ Therefore, graphene is an ideal model structure for the study of hot carrier transport and carrier multiplication, which are inefficient in conventional semiconductor.^[^ ^19^ ^]^ The properties of layered graphene/silicon Van der Waals heterojunctions provide a new structure for exploring hot carrier generation and transport in dynamic semiconductor diodes. Therefore, this study proposes a vertical graphene/silicon DDG, and the detailed hot carrier dynamics of the graphene/silicon interface are explored. To systematically explore the hot carrier dynamic processes of the vertical graphene/silicon DDG, the entire generation, transport, and collection processes of hot carriers are illustrated, which reveal how the hot carriers of graphene in dynamic heterojunction interfaces can be effectively utilized. When the graphene and silicon are in vertical contact, electrons diffuse from the higher Fermi level graphene to the lower Fermi level silicon and form a static depletion region in the microsecond time scale,^[^ ^20^ ^]^ generating a voltage as high as 1.5 V. Following the vertical separation of the graphene and silicon, the dynamic equilibrium of the depletion region is disrupted and diffusion carriers are released and rebound to graphene and silicon, generating an opposite voltage output. The voltage is significantly higher than the barrier height of the graphene/silicon heterojunction. Therefore, hot carriers generated within the rebounding process caused by the built‐in electric field in dynamic graphene/silicon diodes can be collected.

To further enhance the electrical performance of the vertical graphene/silicon DDG, several factors influencing the physical processes are systematically investigated, such as resistivity, work area, and applied force. Compared to metal and silicon substrates, graphene has the unique advantage of an ultralong hot electron lifetime in graphene/silicon Van der Waals heterojunctions,^[^ ^21^ ^]^ which increases the efficiency of hot carrier collection with vertical graphene/silicon DDGs. To further reduce the energy loss of hot carriers, monolayer graphene is applied, achieving a voltage as high as 6.1 V and a current as high as 235.6 nA have been achieved, which provides further evidence for the effective utilization of hot carriers. Specifically, the enhanced voltage is attributed to the ultrafast hot carrier transport in monolayer graphene/silicon Van der Waals heterojunctions and the enhanced current is attributed to the carrier multiplication during the Auger process of graphene. Furthermore, a voltage as high as 17.5 V is achieved by series connection, which is high enough to power portable electronic systems in typical scenarios, indicating the potential of the generator chip inside a self‐powered electronic system through integration design. Although the Van der Waals interaction between the silicon and graphene is strong, the vertical graphene/silicon DDG shows excellent stability and durability, with no obvious voltage attenuation and limited interface damage after working for 1h. This indicates that the vertical graphene/silicon DDG has practical applications with high reliability. This study on the carrier kinetics of a vertical graphene/silicon DDG provides interesting details on DDG electrical properties, highlighting their application as novel and potential in‐situ energy sources for harvesting mechanical energy from the environment. Furthermore, the effective utilization of hot carriers in graphene/silicon DDGs paves the way for further enhancement of the electrical performance of other DDGs.

## 2 Results and Discussion

Graphene has attracted significant attention in the field of electronic devices, especially in wearable devices. In preliminary work, considering high conductive and high mechanical physical properties, a graphene membrane was chosen to fabricate a horizontal dynamic Schottky diode generator with a superior working lifetime and flexibility, which could be used to harvest low‐frequency disordered mechanical motion into electricity. There are two general modes of mechanical motion in nature: horizontal and vertical. Compared to the horizontal mode, the vertical mode is more common in human motion, such as walking and muscle vibration. In order to effectively harvest these low‐frequency disordered vertical mechanical motions and investigate the detailed carrier dynamics processes of a dynamic semiconductor diode, a vertical graphene/silicon dynamic diode was explored. This vertical dynamic semiconductor diode can accurately transfer the irregular, low‐frequency, and weak mechanical force in the vertical direction into electrical energy.

**Figure 1:**
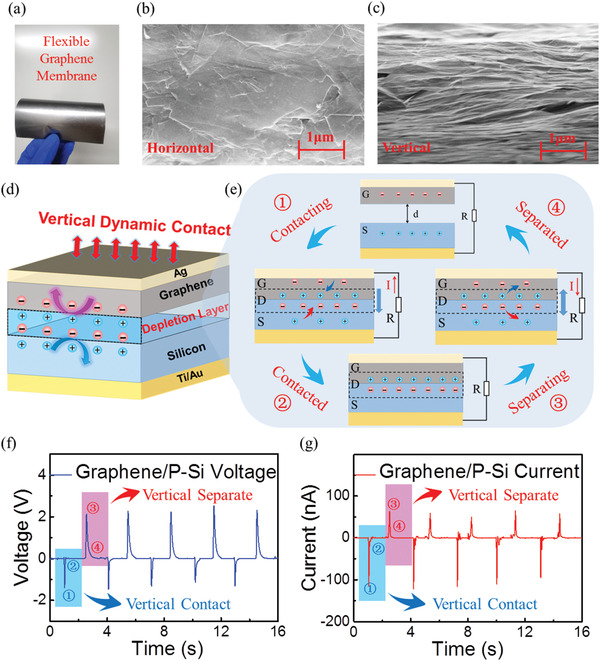
Schematic diagrams and results of vertical graphene/P‐type silicon DDG. a) Image of the large‐scale graphene membrane, which was fabricated with the tape casting method. The SEM images of the graphene membrane in the b) horizontal section and c) vertical section, which show that graphene membrane had heaped layers in the vertical section and deeply linked layers in the horizontal section. The inset scale bar is 1µm. d) Experimental design and detailed 3D structure of the vertical dynamic graphene/silicon heterojunction diode. e) Schematic diagram of the vertical dynamic graphene/silicon heterojunction diode, including the contacting^①^, contacted^②^, separating^③^, and separated^④^ states. f) Voltage over time and g) current over time for the vertical graphene/silicon DDG. Negative and positive voltage/current pulses are the transient voltage/current output during the vertical contact and separate phases, respectively.

As shown in the optical picture in **Figure** **1**a, the graphene membrane has excellent flexibility and mechanical stability, which indicates its superiority for mechanical energy harvesting. Scanning electron microscopy (SEM) images of the graphene membrane were also measured to demonstrate its microstructure in horizontal and vertical sections, as shown in Figure 1b,c. The graphene membrane had heaped layers for the vertical section and deeply linked layers for the horizontal section, which was fabricated by the tape casting method. SEM images of the graphene membrane in a curved state were also measured, as shown in Figure S1 (Supporting Information). During the vertical contact and separate movement of the graphene membrane and silicon substrate, there was no obvious damages to either surface, which ensured the stability and durability of the dynamic graphene/silicon diode, demonstrating its potential application in vertical power harvesting and motion detection.

A detailed 3D diagram and schematic structure of the vertical dynamic graphene/silicon heterojunction diode are illustrated in Figure 1d. The graphene membrane was in vertical dynamic contact with the semiconductor silicon substrate under a fixed external pressure. As shown in Figure S2 (Supporting Information), the thickness of the graphene membrane was 40.12 µm. With the contact of graphene and P‐type silicon, a depletion layer was formed in the graphene/silicon interface. The detailed carrier dynamics process of the vertical dynamic diode is illustrated in Figure 1e, including the contacting, contacted, separating, and separated states. First, before the graphene membrane connected with P‐type silicon, the majority carriers of graphene and P‐type silicon were electrons and holes, respectively. Then, the distance between the graphene membrane and P‐type silicon decreased from 0.2 to 0 mm under the external force. According to the continuity equation, the diffused electrons and holes will diffuse across the interface and have potential to reach electrodes due to the carrier concentration difference between silicon and graphene, ultimately generating current in the external circuit.^[^ ^22^ ^]^ With the establishment of the equilibrium between the diffusion and drift currents in the graphene/silicon van der Waals heterojunction, an electron depletion region was formed in the surface of P‐type silicon substrate. Therefore, in the contacting state, a voltage as high as 1.5 V and a current as high as 122.3 nA from silicon to graphene were achieved, as shown in the vertical contact section in Figure 1f,g.

After the graphene membrane completely contacted with the P‐type silicon substrate, a stable depletion layer was established at the interface of the graphene/silicon heterojunction without an external current, in the contacted state. Then, the graphene membrane separated with p‐type silicon under the external force, the electron depletion region disappeared at the surface of the P‐type silicon substrate, and the electrons and holes diffused back to the graphene and silicon substrate, forming a positive pulse voltage/current. In this separating state, a voltage as high as 2.1 V and a current as high as 64.3 nA from graphene to silicon were achieved, as shown in the vertical separate section in Figures 1f,g. Finally, when the distance between the graphene membrane and silicon substrate increased to 0.2 mm, the electrons and hole of the graphene and P‐type silicon stabilized under the Fermi level difference again, as shown in the separated state. With the periodic contact‐separate movement of the graphene membrane under the action of external mechanical force, a vertical graphene/silicon DDG with stable pulse voltage and current as high as 2.1 V and 122.3 nA, respectively, were achieved, as shown in Figure 1f,g.

**Figure 2:**
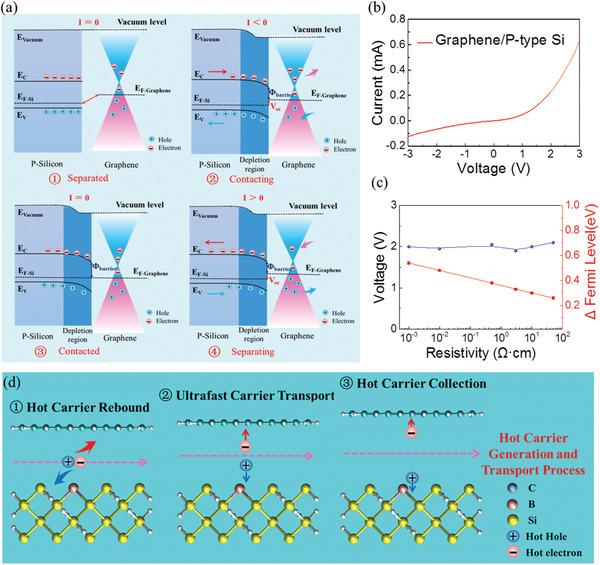
Physical mechanisms of the vertical graphene/P‐type silicon DDG. a) Carrier distribution and energy band diagram of the vertical graphene/silicon DDG when the graphene and silicon were in the separated^①^, contacting^②^, contacted^③^, and separating^④^ states. b) *I–V* curve of the vertical graphene/silicon heterojunction DDG in a quasi‐dynamic equilibrium state from ‐3 to 3 V. c) Voltage outputs of the vertical graphene/silicon DDG with different resistivity of 0.001, 0.01, 0.5, 3, 10, and 50 Ω cm. d) Hot carrier generation and transport process diagram of the vertical graphene/silicon DDG when the graphene and silicon were in contact.

In order to further explore the physical mechanisms of the vertical graphene/silicon DDG in a systematic semiconductor theoretical framework, the entire establishment, quasi‐dynamic equilibrium, and destruction processes of the interface depletion layer were explored, which played a key role in electricity generation. The work functions of graphene and P‐type silicon were 4.60 and 5.12 eV, respectively. Therefore, the Fermi level of the P‐type silicon substrate was lower than the graphene membrane, and the carrier distribution and energy band diagram before graphene and silicon contacting were shown in the separated state in **Figure** **2**a. When the graphene membrane and silicon substrate were gradually contacting, electrons diffuse from the conduction band of the graphene membrane to the silicon and the holes diffused from the valence band of P‐type silicon to the graphene, generating a current from the silicon to the graphene membrane. The Fermi level difference between graphene and silicon gradually decreased and a depletion layer with potential barrier was established in the graphene/silicon interface, in the contacting state.

After the graphene membrane and silicon substrate were in stable contact, a quasi‐dynamic equilibrium was established in the graphene/silicon heterojunction, where the diffusion and drift current cancelled each other out. In this dynamic balanced heterojunction, no current flowed through the heterojunction with the external circuit, where the energy band structure was shown in the contacted state. However, when the graphene membrane and silicon substrate were separated under mechanical force, the diffused electrons/holes that had been trapped in the space charge area of heterojunction were released and rebounded to the graphene membrane/silicon through the external circuit, generating a current from the graphene membrane to the silicon substrate. The detailed carrier distribution and energy band diagram of the dynamic graphene/silicon diode in this separating process is shown in the separating state. In the repeatable contact and separate cycles of the vertical graphene/silicon DDG, the electrons and holes in the graphene membrane and silicon substrate were diffused and rebounded between the graphene and silicon with the dynamic equilibrium of the depletion region. In contrast to the traditional triboelectric nanogenerator based on a displacement current, a conduction current across the graphene/silicon heterojunction was involved in generating power, leading to an ultrafast response time.^[^ ^23^ ^]^ Figure 2b shows the *I*–*V* curve of the vertical graphene/silicon heterojunction DDG in the quasi‐dynamic equilibrium state, indicating the formation of the depletion region during the contact of the graphene membrane and silicon substrate, which can achieve ideal rectification characteristics with limited leakage under a negative bias voltage.

Furthermore, to quantitatively reveal the relationship between the voltage output and the Fermi level difference, as well as the interface barrier of the dynamic graphene/silicon diode, a series of P‐type silicon substrates with different resistivity of 0.001, 0.01, 0.5, 3, 10, and 50 Ω cm were explored. The Fermi level of the P‐type silicon was calculated using the following equations:^[^ ^24^ ^]^

(1) $$\sigma =\frac{1}{\rho }\approx qp\mu_{p}$$

(2) $$E_{F-s}\approx E_{i}-k_{B}T\ln\frac{p}{n_{i}}$$

where *σ* and *ρ* are the conductivity and resistivity of the P‐type silicon substrate, respectively. *μ*_*p*_ is the intrinsic hole mobility of the silicon, and *q* is the quantity of the electric charge. Therefore, the carrier concentration of P‐type silicon was calculated using equation (1). *E*_i_ is the median of the conduction and valence bands, *k*_B_ is the Boltzmann constant, *T* is the temperature, and *n_i_* is the intrinsic electron concentration of the silicon. For P‐type silicon, the intrinsic hole mobility *μ*_*p*_ was 500 cm^2^/Vs, the intrinsic electron concentration *n_i_* was 1.5 × 10^10^ cm^−3^. The conduction and valence bands of silicon were 4.05 and 5.17 eV, respectively. Therefore, the *E_i_* can be calculated as 4.61 eV.^[^ ^24^ ^]^ *E*_*F* − *s*_ is the Fermi level of the P‐type silicon substrate, which was calculated using equation (2). As shown in Figure 2c, the Fermi levels of the P‐type silicon were calculated as 5.14, 5.08, 4.98, 4.93, 4.90, and 4.86 eV with the different resistivity of 0.001, 0.01, 0.5, 3, 10, and 50 Ω cm, respectively. However, the voltage outputs of the vertical graphene/silicon DDG with different resistivity were always ≈2.0 V, which was significantly larger than the Fermi level difference between graphene and silicon, which was as high as 0.54, 0.48, 0.38, 0.33, and 0.26 eV.

This high voltage response was attributed to the hot carrier generation and transport between the vertical dynamic graphene/silicon diode, especially in the graphene layer. As shown in Figure 2d, the space carrier especially the diffused electrons and holes in the depletion region of the dynamic graphene/silicon heterojunction was rebound by the ultrahigh built‐in electric field, generating hot carriers with higher energy than the barrier height of the heterojunction. Then, due to the ultrafast carrier transport in the dynamic graphene/silicon Van der Waals heterojunction, those hot carriers were transmitted to the graphene membrane in a microsecond time scale.^[^ ^20^ ^]^ Finally, those hot carriers were effectively collected in the graphene membrane with an ultralong hot electron lifetime. In comparison, the horizontal graphene/silicon DDG generated a constant voltage under continuous movement, but this was significantly lower than the vertical graphene/silicon DDG, as shown in Figure S3 (Supporting Information). The voltage difference was attributed to the defect recombination of carriers in the interface caused by the continuous movement, which can be decreased through further friction reduction. The detailed energy band diagram of the horizontal graphene/silicon DDG is shown in Figure S4 (Supporting Information).

**Figure 3:**
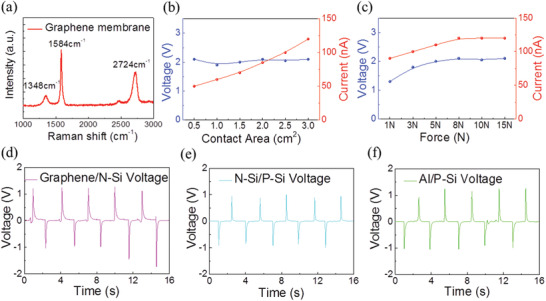
Electrical properties of the vertical DDG. a) Raman spectrum of the graphene membrane at a room temperature of 25 °C. b) Voltage and current output of the vertical graphene/P‐type silicon DDG with different work areas. c) Voltage and current output of the vertical graphene/P‐type silicon DDG with different applied forces on the graphene membrane. Voltage outputs of the vertical d) graphene/N‐type silicon, e) Al/P‐type silicon, and f) N‐type/P‐type silicon DDGs.

In the hot carrier generation and transport processes, the graphene membrane plays a key role in hot carrier transport and collection. The Raman spectrum of the graphene membrane at a room temperature of 25°C was measured, as shown in **Figure** **3**a. There were three peaks in this in‐situ Raman spectra: D‐peak (1348 cm^−1^), G‐peak (1584 cm^−1^), and 2D‐peak (2724 cm^−1^). The low intensity ratio between the 2D‐peak and G‐peak indicates the graphene membrane was multilayer. In particular, the weak D‐peak showed the high quality and limited crystal defects of the graphene membrane,^[^ ^25^ ^]^ which contributed to the limited carriers recombination and effective collection of the rebounding hot carriers in the vertical graphene/silicon DDG. To systematically explore the electrical properties of the vertical graphene/silicon heterojunction DDG, the voltage and current with different contact areas or forces were measured. As shown in Figure 3b, the current was positively proportional to the contact area, but the voltage remained almost unchanged, indicating that the voltage depended on the energy level of hot carriers but the current depended on the quantity of hot carriers generated at the interface. A voltage up to 2.1 V and current up to 123.6 nA were achieved under the contact area of 3.0 cm^2^, which can be further improved by interface array design.

Furthermore, the relationship between the voltage and current output with different applied forces was also explored, as shown in Figure 3c. The voltage and current output were positive related with the force applied in the graphene membrane until good heterojunction characteristics were formed between the graphene membrane and silicon. It was found that the maximal voltage of 2.1 V and current of 123.6 nA were achieved under a force of 8 N, where the dynamic graphene/silicon heterojunction exhibited the optimal characteristics. In addition, the electrical performance of the vertical graphene/silicon DDG under illumination was also studied to eliminate the influence of environmental light, as shown in Figure S5 (Supporting Information). As mentioned above, the high voltage output was related to the hot carriers in the graphene membrane, where the carrier density and Fermi level could be adjusted by the doping method. Therefore, FeCl_3_ was selected as an intercalation of the graphene membrane, which could export the electrons in the graphene layer. As shown in the Raman spectrum of the graphene membrane with FeCl_3_ doping in Figure S6 (Supporting Information), the intercalation of FeCl_3_ resulted in a P‐type doping and downward Fermi level for the graphene. This P‐type doping of graphene decreased the barrier height of the graphene/silicon heterojunction, leading to a directional and quantitative adjustment of the voltage output through further changes in concentration, as shown in Figure S7 (Supporting Information).

To address the universality of this vertical dynamic diode generator, a series of different semiconductor structure diodes were also measured. Compared with the vertical graphene/P‐type silicon DDG, an opposite voltage direction was observed for the vertical graphene membrane/N‐type silicon DDG, as the Fermi level of the graphene membrane was lower than the N‐type silicon. Figure S8 (Supporting Information) demonstrates the schematic diagram of the vertical graphene/N‐type silicon DDG. As shown in Figure 3d, the voltage of 1.8 V achieved during the contact and separate stages was also significantly higher than the Fermi level difference between the graphene and N‐type silicon, which also verified the hot carrier transport process in the vertical graphene/N‐type silicon DDG. However, this voltage was smaller than the voltage of the vertical graphene/P‐type silicon DDG and exhibited fluctuations during cyclic movement, indicating energy loss and carrier recombination during the hot carrier transport in the vertical graphene/N‐type silicon DDG. As the work function difference between graphene/N‐type silicon was smaller than graphene/P‐type silicon, the built‐in electric field intensity of graphene/N‐type silicon was smaller. Furthermore, the type of the rebounded carriers of vertical graphene/N‐type silicon and graphene/P‐type silicon were electrons and holes, respectively. Therefore, the vertical graphene/P‐type silicon DDG can fully utilize the ultrahigh electron mobility of graphene, limiting energy loss and carrier recombination during the hot carrier transport.

In addition to the dynamic graphene/silicon heterojunction, vertical Al/P‐type silicon and N‐type/P‐type silicon DDGs were explored. As shown in Figure 3e,f, a voltage of ≈1.0 V was achieved in the vertical Al/P‐type silicon and N‐type/P‐type silicon DDGs, which was similar to the Fermi level difference value between the respective diodes. As the Fermi level of P‐type silicon was also lower than Al and N‐type silicon, the direction of the voltage output during the contact and separate stages was the same as for the graphene membrane. However, the voltage output of the vertical Al/P‐type silicon and N‐type/P‐type silicon DDGs was much lower than the former vertical graphene/P‐type silicon DDG. It follows that the hot carriers in the metal film or semiconductor substrate were limited by the degradation of energy during the transport process. Therefore, the voltage output was on the verge of the barrier height value of the dynamic diode. Compared with Al and N‐type silicon, the hot carriers with ultralong lifetime in graphene membrane can effectively transfer and collect with limited attenuation, leading to a significantly higher voltage output.

**Figure 4:**
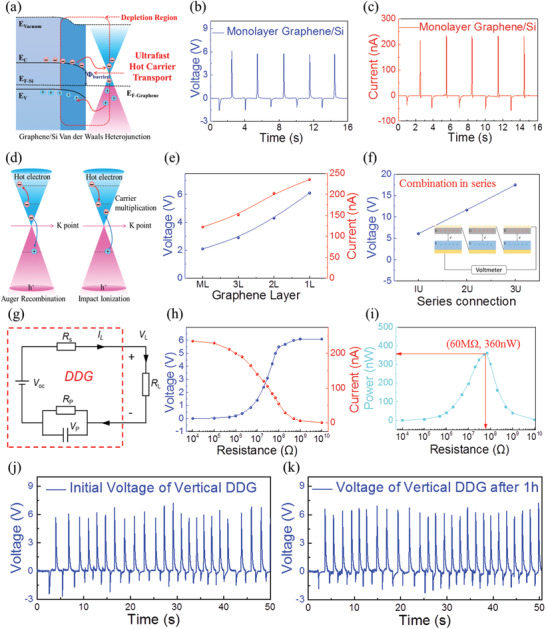
Optimized electrical performance of vertical monolayer graphene/silicon structure DDG. a) Ultrafast hot carriers transport process diagram of the graphene/silicon Van der Waals heterojunction in the depletion region. b) Voltage output and c) current output of the vertical monolayer graphene/silicon DDG. d) Linear energy dispersion of graphene around the K point, including the Auger recombination and impact ionization of hot carriers. e) Voltage and current outputs of the vertical graphene/silicon DDG with different layers of graphene. f) Voltage output of vertical graphene/silicon DDG unit combined in one, two and three series. g) Equivalent circuit of vertical graphene/silicon DDG to measure the power output under different load resistances. h) Work voltage and work current of the vertical graphene/silicon structure DDG as a function of the electrical load resistance. i) Calculated corresponding power output as a function of electrical load resistance, with a peak power of 360 nW and a load resistance of 60 MΩ. j) Initial voltage of vertical graphene/silicon DDG. k) Voltage of vertical graphene/silicon DDG after working for 1 h.

To optimize the electrical performance and further develop the detail hot carrier transport mechanism of the vertical graphene/silicon DDG, few‐layer graphene was applied. As shown in **Figure** **4**a, due to the ultrafast carrier transport of the graphene/silicon heterojunction, hot carriers generated in the depletion region were effectively collected by the graphene layer, especially the monolayer graphene with excellent carrier mobility. Figure 4b shows the voltage output of the vertical monolayer graphene/silicon DDG. It can be seen that a voltage as high as 6.1 V was achieved in monolayer graphene device, which is approximately three times larger than the multilayer graphene membrane, indicating the limited energy loss of hot carriers in monolayer graphene. Furthermore, the current of the vertical monolayer graphene/silicon DDG was measured as high as 235.6 nA, which is approximately two times larger than the multilayer graphene membrane, as shown in Figure 4c. The enhanced voltage output was attributed to the limited energy loss under ultrafast carrier transport of the graphene/silicon heterojunction, and the enhanced current output was attributed to the carrier multiplication in the Auger process of graphene.

As shown in Figure 4d, there were two types of Auger process in graphene: Auger recombination and impact ionization. In Auger recombination process, the electron was scattered from the conduction band to the valence band, while energy was transferred to the other electron in conduction band, generating hot carriers with a higher state within the conduction band. Following with the electron relaxing to the energetically lower state during the impact ionization process, the electron in the valence band was excited into the conduction band, which was efficient enough induce a significant carrier multiplication, despite the competition between Auger recombination and other relaxations. This reveals how hot electrons and holes of the DDG interface generate and evolve in the graphene layer. The enhanced voltage and current output of the monolayer graphene/silicon DDG proved that more hot carriers with higher energy were excited owing to the Auger process in monolayer graphene. In a conventional semiconductor structure DDG, this Auger process is always suppressed by energy and momentum conservation limitations, which are difficult to satisfy simultaneously owing to the bandgap and energy dispersion in the traditional semiconductor substrate.

In addition to the investigation of the monolayer graphene/P‐type silicon DDG, different layers of graphene were also studied. Bilayer, trilayer and thin graphene film were used to perform the same contact and separate experiments. As shown in Figure 4e, the voltage and current output of thin film, trilayer, bilayer, and monolayer graphene based DDGs were 2.1, 2.9, 4.3, and 6.1 V and 122.3, 151.1, 202.6, and 235.6 nA, respectively. With the decrease in layer numbers, the voltage and current output generated by the graphene/silicon DDG increased, which was each larger than the interface barrier height and Fermi level difference of the graphene/silicon heterojunction, further proving the proposed mechanism of hot carrier generation and transport in graphene. Therefore, the graphene has a unique advantage over metal films or other semiconductors in the construction of dynamic diodes, in which hot carriers can be effectively utilized due to the Auger process and the ultra‐long hot electron lifetime of graphene. The vertical graphene/silicon DDG provides an ideal model structure to explore hot carrier dynamics in graphene. Furthermore, the voltage output can be easily amplified by series connection. As shown in Figure 4f, two and three graphene/silicon DDGs combined in series generated large voltage output of 11.6 and 17.5 V, respectively. The enhanced voltage output indicates the linear superposition of different DDG units, which is sufficient for powering frequently used electronic systems, indicating its potential as generator chip through integration design.

Finally, to quantitatively demonstrate the power output of the vertical graphene/silicon DDG, the work voltage (*V*_L_) and work current (*I*_L_) outputs as a function of electrical load resistance (*R*_L_) were measured, as shown in Figure 4g. The internal resistance (*R*_S_) was calculated with the different load resistance matching method, in which the power output achieved a maximum peak. The corresponding power output was controlled by the work voltage and work current, which was calculated as a product of the voltage and current. As shown in Figure 4h, an increasing voltage and decreasing current were achieved with an increase in external load resistance. The maximum voltage and current output of the vertical dynamic graphene/silicon diode reached 6.1 V and 235.6 nA, respectively. The calculated maximum work power output approached 360 nW with a load resistance of 60 MΩ, with an internal resistance of the vertical graphene/silicon DDG of ≈60 MΩ, as shown in Figure 4i. With the advantage of a high electrical performance, the vertical graphene/silicon DDG has a high potential for applications in real‐world scenarios, such as the recycling of mechanical energy in vehicles and the conversion of electrical energy in wearable devices.

Stability and durability are key parameters for DDGs in real‐world applications. Therefore, experiments were conducted to establish the relationship between the voltage outputs of the vertical graphene/silicon DDG and the working time. As shown in Figure 4j, the initial voltage of the vertical graphene/silicon DDG was as high as 6.1V, and the voltage fluctuation between different periods of vertical movement was limited. After working for 1 h, the voltage of the vertical graphene/Si DDG was still as high as 6.1V, with no obvious attenuation compared with the initial voltage, as shown in Figure 4k. This was attributed to the good adhesion between graphene and the insulator substrate, alongside negligible abrasion during the vertical movement process. As graphene is a layered material with excellent flexibility, no obvious damage was observed in the surface of the graphene membrane during vertical movement for 1 h, as shown in Figure S9 (Supporting Information). The continuous voltage output of the vertical graphene/silicon DDG shows excellent repeatability at ≈6.1 V, as shown in Figure S10 (Supporting Information). Therefore, the vertical graphene/Si DDG has potential practical applications with high reliability.

## 3 Conclusion

In summary, a vertical graphene/silicon DDG was demonstrated as a potential approaches for meeting the energy demands of modern society. Compared with previously developed DDGs, this vertical graphene/silicon DDG can effectively collect hot carriers generated by the built‐in electric field during instant contact and separation between the graphene and silicon, due to the ultralong hot electron lifetime of graphene and ultrafast carrier transport at the dynamic diode interface. In particular, a high voltage of 6.1 V and current of 235.6 nA were achieved in the monolayer graphene/silicon DDG unit. The enhanced electrical performance was attributed to the ultrafast carrier transport and hot carrier multiplication during the Auger process of graphene. A voltage as high as 17.5 V was achieved by series connection, which is sufficiently high to power frequently used electronic systems, indicating its potential as power generation chip through further integration design. This study provides a novel and potential in‐situ energy source for harvesting mechanical energy from the environment.

## 4 Experimental Section

### Device Fabrication

The graphene membrane was fabricated with the tape casting method, in which the few‐layer graphene powder was dispersed in water uniformly by the ultrasonic method, before heating and drying in a PET film. The ohmic electrode of the graphene membrane was fabricated with silver epoxy and drying with hot plate at a temperature of 110 °C. Then, a plain conductor was connected to the silver electrode before being sealed using polydimethysiloxane (PDMS) to avoid short circuiting. The thickness of the graphene membrane and the Al film was 40 µm. Furthermore, single side polished N‐type and P‐type doped silicon substrates were used, which were dipped into 10 wt% HF for 5 min to remove the native silicon oxide layer. The resistivity of P‐type and N‐type silicon was 0.5 Ω cm. The Ti/Au (5 nm/50 nm) electrode of the N‐type or P‐type silicon was grown using the magnetron sputtering method on the unpolished back side, which formed an ohmic contact after annealing in inert gas at a temperature of 400 °C. The monolayer, bilayer, and trilayer graphene were grown using the chemical vapor deposition (CVD) method and then transferred to a PET substrate. The graphene membrane was contacted and separated with the silicon substrate by a constant external force, ensuring a uniform surface contact in the vertical graphene/silicon DDG.

### Characterization Analysis and Measurement

The image of the graphene membrane was recorded using a high definition camera. SEM images of the graphene membrane were recorded using a HITACHI S‐4800 system. Keithley 2400 and Agilent B1500A systems were used to record the current–voltage (*I*–*V*) data. A Keithley 6514 system electrometer and DMM6500 multimeter were used to record the real time voltage and current responses of the vertical graphene/silicon DDG, which were controlled by a LabView‐based data acquisition system with a sampling rate of 10 000 s^−1^. The Raman spectrum of the graphene membrane was measured using the Renishaw inVia Reflex under an excitation laser (532 nm, 1 mW), which was focused on a circle with a radius of 1 mm.

## Conflict of Interest

The authors declare no conflict of interest.

## Author Contributions

S.L. designed the experiments, analyzed the data, and conceived the study. Y.L. designed and carried out the experiments, discussed the results, and wrote the paper. R.S., X.Y., Y.Y., H.Z. Y. Y., C.L., Z.Y., L.F., and L.L. discussed the results and assisted with experiments. All authors contributed to the preparation of the manuscript.

## Supporting information

Supporting Information

## Data Availability

The data that support the findings of this study are available from the corresponding author upon reasonable request.
