# Supplementary material for: Hot Carrier Transport and Carrier Multiplication Induced High Performance Vertical Graphene/Silicon Dynamic Diode Generator
Source: Adv Sci (Weinh). 2022 May 23;9(21):2200642. doi: 10.1002/advs.202200642 (PMC9313483; doi:10.1002/advs.202200642)
Supplement: Supplementary file 1 — Supporting Information [file ADVS-9-2200642-s001.pdf]

Supplementary Materials for

Hot Carrier Transport and Carrier Multiplication Induced High Performance Vertical Graphene/Silicon Dynamic Diode Generator

Yanghua Lu, Runjiang Shen, Xutao Yu, Deyi Yuan, Haonan Zheng, Yanfei Yan, Chang Liu, Zunshan Yang, Lixuan Feng, Linjun Li and Shisheng Lin**

Dr Y. -H. Lu, R. -J. Shen, X. -T. Yu, D. -Y. Yuan, H. -N. Zheng, C. Liu, Z. -S. Yang, L. -X. Feng, Prof. S. -S. Lin

College of Microelectronics, College of Information Science and Electronic Engineering, Zhejiang University, Hangzhou, 310027, P. R. China

Email: luyanghua6@zju.edu.cn; shishenglin@zju.edu.cn

Prof. L. -J. Li, Prof. S. -S. Lin

State Key Laboratory of Modern Optical Instrumentation, Zhejiang University, Hangzhou, 310027, P. R. China

*Corresponding author.

This file includes:

Figs. S1 to S10

Figure S1: Optical and SEM images of graphene membrane under in a curved state.

Figure S2: SEM image of graphene membrane in vertical section.

Figure S3: Schematic diagram and corresponding voltage output of the horizontal graphene/silicon DDG under the continuous movement.

Figure S4: Energy band diagram of the horizontal graphene/silicon DDG under the continuous movement.

Figure S5: Electrical performance of vertical graphene/silicon DDG under illumination.

Figure S6: Raman spectrum of the graphene membrane with and without $FeCl_3$ doping at room temperature of 25℃.

Figure S7: Energy band diagram and corresponding voltage output of the vertical graphene/silicon DDG with $FeCl_3$ doping.

Figure S8: Schematic diagram of the vertical dynamic graphene/N-type silicon heterojunction diode.

Figure S9: SEM images of the graphene membrane in vertical dynamic graphene/silicon DDG before and after working for 1 h.

Figure S10: Continuous voltage output of vertical graphene/silicon DDG for 300s.

Figure S1.

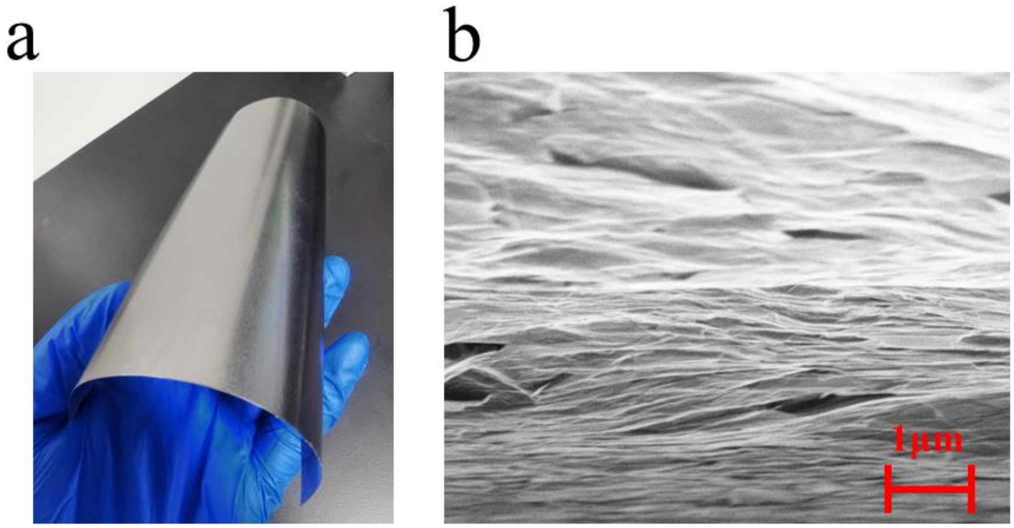

Figure S1. Optical and SEM images of graphene membrane under in a curved state. The inset scale bar is 1μm. It can be found that the graphene membrane used is heaped up layer by layer in vertical section. And no obvious damages are introduced under the bend of graphene membrane, indicating its excellent flexibility and mechanical stability, which ensures the working lifetime and stability of the dynamic graphene/silicon diode.

Figure S2.

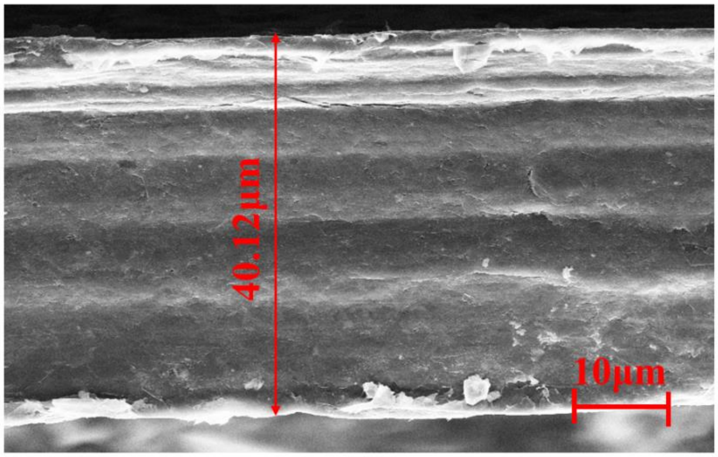

Figure S2. SEM image of graphene membrane in vertical section. The inset scale bar is 10μm. The thickness of graphene membrane used here is 40.12 μm.

Figure S3.

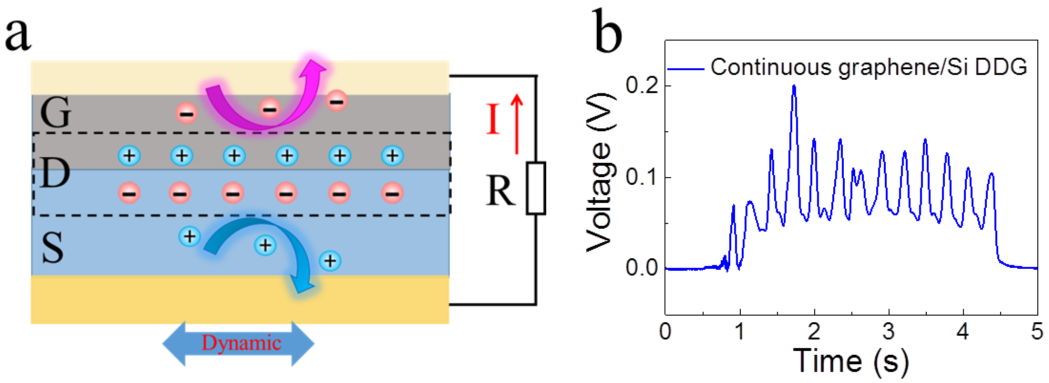

Figure S3. Schematic diagram and corresponding voltage output of the horizontal graphene/silicon DDG under the continuous movement. In this horizontal graphene/silicon DDG, a constant voltage as high as 0.2 V can also achieved under the continuous movement, but much lower than the above mentioned vertical graphene/silicon DDG.

Figure S4.

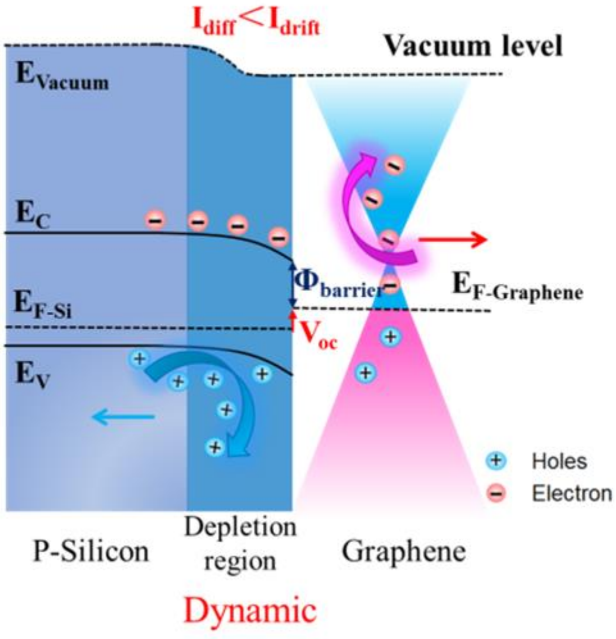

Figure S4. Energy band diagram of the horizontal graphene/silicon DDG under the continuous movement. In this horizontal graphene/silicon DDG, the voltage output is much lower than the above mentioned vertical graphene/silicon DDG. We attribute the voltage decrease to the defect recombination of carriers under the continuous movement, which can be decrease through further reducing friction.

Figure S5.

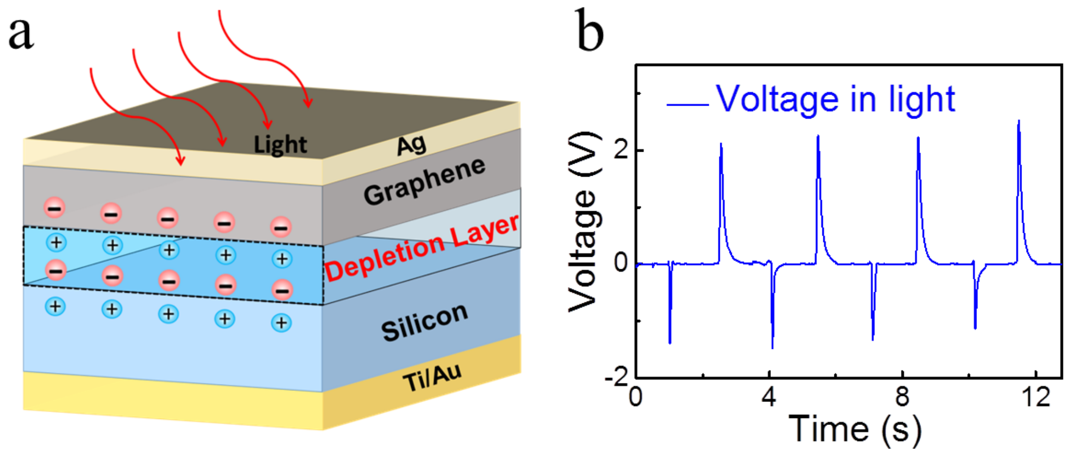

Figure S5. Electrical performance of vertical graphene/silicon DDG under illumination. It can be found that environmental light has limited influence to the electrical performance of vertical graphene/silicon DDG.

Figure S6.

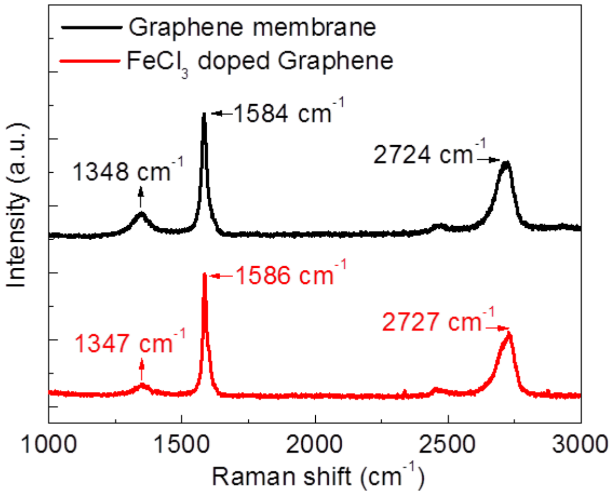

Figure S6. Raman spectrum of the graphene membrane with and without $FeCl_3$ doping at room temperature of 25℃. As $FeCl_3$ being chosen as intercalation of graphene membrane, the electrons in $FeCl_3$ can transfer to the graphene layer. The red shift of Raman peak indicates the P-type doing of graphene.

Figure S7.

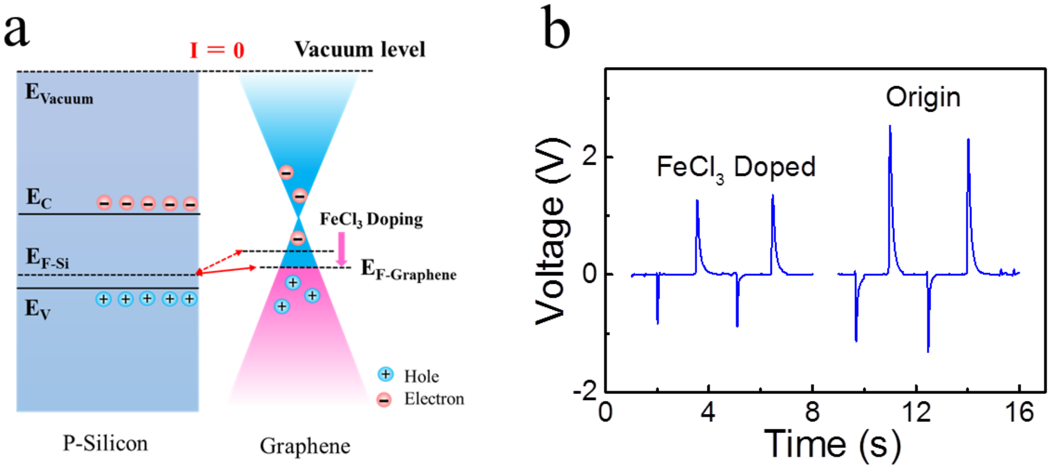

Figure S7. Energy band diagram and corresponding voltage output of the vertical graphene/silicon DDG with $FeCl_3$ doping. The intercalation of $FeCl_3$ leads a P-type doing of graphene and downward Fermi level of graphene. This P-type doping of graphene will decrease the barrier height of graphene/silicon heterojunction, leading a decrease of voltage output.

Figure S8.

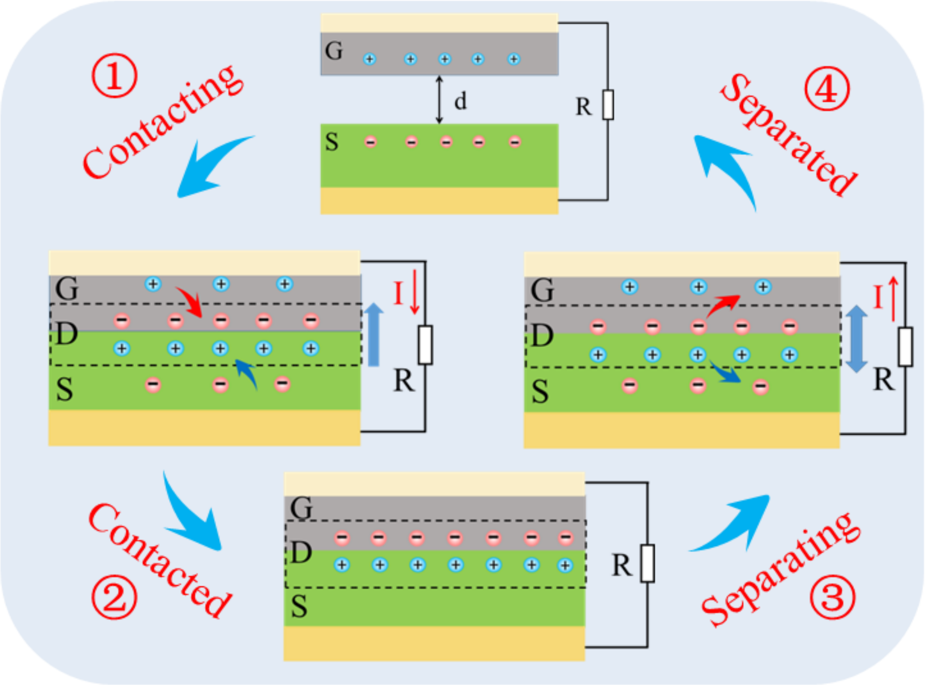

Figure S8. Schematic diagram of the vertical dynamic graphene/N-type silicon heterojunction diode, including the contacting state①, contacted state②, separating state③ and separated state④. Compared with the above mentioned vertical graphene/P-type silicon DDG, Fermi level of graphene membrane is lower than the N-type silicon, so the voltage direction of vertical graphene membrane/N-type silicon DDG is opposite with the vertical graphene/P-type silicon DDG.

Figure S9.

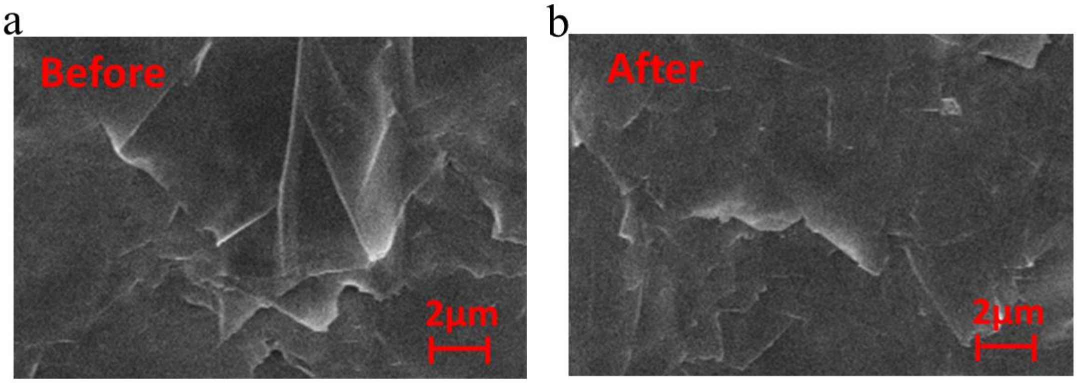

Figure S9. SEM images of the graphene membrane in vertical dynamic graphene/silicon DDG (a) before and (b) after 1 hour working. Although the Van der Waals interaction between the silicon and Ggraphene is strong, the vertical graphene/silicon DDG shows excellent stability and limited damage to graphene layer. As graphene is a layered material with excellent flexibility, no obvious defect of damage has been involved in the surface of graphene membrane.

Figure S10.

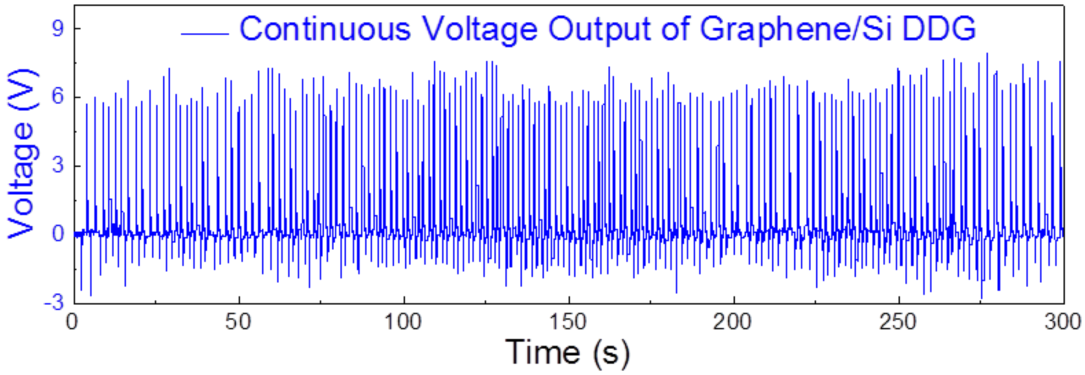

Figure S10. Continuous voltage output of vertical graphene/silicon DDG for 300s. As graphene is graphene is a layered material with excellent flexibility, the continuous voltage output of vertical graphene/silicon DDG shows excellent repeatability around 6.1 V.
